# A modified SEIR model applied to the data of COVID-19 spread in Saudi Arabia

**DOI:** 10.1063/5.0029698

**Published:** 2020-12-04

**Authors:** Hamdy M. Youssef, Najat A. Alghamdi, Magdy A. Ezzat, Alaa A. El-Bary, Ahmed M. Shawky

**Affiliations:** 1 Mechanical Engineering Department, College of Engineering and Islamic Architecture, Umm Al-Qura University, Makkah 21955, Saudi Arabia; 2 Department of Mathematics, Faculty of Applied Science, Umm Al-Qura University, Makkah 21955, Saudi Arabia; 3 Department of Mathematics, College of Science and Arts, Qassim University, Al Bukairiyah, Al Qassim, 52725, Saudi Arabia; 4 Basic and Applied Science Institute, Arab Academy for Science, Technology, and Maritime Transport, P.O. Box 1029, Alexandria, Egypt; 5 Science and Technology Unit (STU), Umm Al-Qura University, Makkah 21955, Saudi Arabia

## Abstract

The Susceptible-Exposed-Infectious-Recovered (SEIR) model is an established and appropriate approach in many countries to ascertain the spread of the coronavirus disease 2019 (COVID-19) epidemic. We wished to create a new COVID-19 model to be suitable for patients in any country. In this work, a modified SEIR model was constructed. We used the real data of COVID-19 spread in Saudi Arabia for statistical analyses and complex analyses. The reproduction number and detailed review of stability demonstrated the complexities of our proposed SEIR model. The solution and equilibrium condition were explored based on Jacobian’s linearization approach to the proposed SEIR model. The state of equilibrium was demonstrated, and a stability study was conducted in the disease-free environment. The reproduction number was measured sensitively against its internal parameters. Using the Lyapunov principle of equilibrium, the overall consistency of balance of our model was demonstrated. Findings using the SEIR model and observed outcomes due to COVID-19 spread in Saudi Arabia were compared. The modified SEIR model could enable successful analyses of the spread of epidemics such as COVID-19. An “ideal protocol” comprised essential steps to help Saudi Arabia decelerate COVID-19 spread. The most important aspects are to stay at home as much as possible and for infected people to remain in an isolated zone or secure area.

## INTRODUCTION

The human immunodeficiency virus (HIV) is a species of *Lentivirus* that infects humans. Over time, HIV infection causes acquired immunodeficiency syndrome (AIDS). Until 1980, AIDS was not recognized and the link with HIV dissemination not made. After 1980, five continents were ravaged by AIDS, with >300 000 individuals diagnosed during this period.^1^

Ebola virus disease (EVD) is a viral hemorrhagic fever of humans and other primates caused by ebolaviruses. In recent years, EVD has killed many people worldwide. Some researchers have postulated that it was transmitted from infected animals (e.g., bats). Due to the contact between various animals and humans, several deadly diseases can occur.

Since December 2019, healthcare systems worldwide have been struggling with management of the coronavirus disease 2019 (COVID-19) pandemic. As of 31 October 2020, nine million people worldwide have been diagnosed with COVID-19, and the number of cases is increasing daily in USA, UK, and mainland Europe. The origin of the virus that causes COVID-19, severe acute respiratory syndrome coronavirus-2 (SARS-CoV-2), is not known. The incubation period of SARS-CoV-2 is 10–14 days. A vaccine against SARS-CoV-2 infection has not been developed, though clinical trials in several countries are underway.

Management of highly infectious diseases (e.g., AIDS, EVD, and COVID-19) is reliant primarily on rapid detection and isolation of infected individuals. The movement of infectious individuals from location-to-location affects other people and triggers disease spread. During the current COVID-19 pandemic, international travel has been reduced significantly.^2^ Scientists and researchers worldwide are trying to find a vaccine or cure for COVID-19.

An epidemic can be identified and interpreted through statistical simulations. Several statistical models for specific diseases and pathogens have been established.^3,4^ From 31 December 2019 to 28 January 2020, Wu and co-workers introduced the Susceptible-Exposed-Infectious-Recovered (SEIR) model.^5^ Read and co-workers reported a reproductive number (RN) for COVID-19 of 3.1 based on data fitting for the SEIR model using an assumption of Poisson-distributed daily time increments.^6^ Tang and co-workers proposed a deterministic compartmental model that included progression of clinical disease, individual epidemiological status, and participant behavior.^7^ They noted that intervention methods (e.g., exclusion of infected individuals) accompanied by quarantine could reduce the likelihood of transmission and reproduction of SARS-CoV-2.

To assess the size of the COVID-19 outbreak in Wuhan (Hubei Province, China), Imai and co-workers carried out computational models of a potential epidemic focusing on human transmissions. Their findings suggested that intervention could block >60% of SARS-CoV-2 transmissions to avoid outbreaks.^8^ Gao and co-workers developed an in-depth algorithm for evaluating and forecasting the infectivity of SARS-CoV-2. They suggested that the hosts of SARS-CoV-2 could be bats and minks.^4^

Most statistical models have highlighted the vital role of the direct-transmission pathway between humans in the COVID-19 pandemic. This has been shown by the fact that (i) most infected persons in Wuhan had no relationship with the wet market that was thought be the source of SARS-CoV-2 infections; (ii) the number of infections increased rapidly; and (iii) COVID-19 spread to all provinces in China.^2,5,9–13^ People suffering from COVID-19 can be asymptomatic but pass on SARS-CoV-2 infection to others through close interactions. Statistical models have not considered the effect of the climate on COVID-19. Other modeling studies for the COVID-19 pandemic have been carried out.^1,3,4,6,11,13–23^

A mathematical model can draw definite and detailed conclusions about the COVID epidemic. Hence, a cascade of SEIR models has been established to explain the transmission mechanisms from the source, storage reservoir, and hosts for humans.^18,23,24^

We wished to create a new COVID-19 model to be suitable for patients in any country. In this work, a modified SEIR model was constructed. We aimed to discover the dynamics of SARS-CoV-2 transmission to humans. We also wished to determine the ideal protocols, control, and strategies that can reduce the outbreak significantly based on data on COVID-19 spread in Saudi Arabia.

## METHODS

### Formulation of a modified SEIR model

**FIG. 1.**
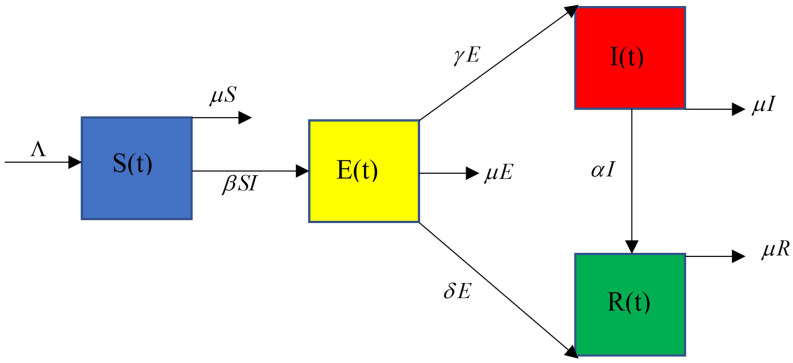
Flowchart of the proposed SEIR model.

During COVID-19 spread in any country, the population can be divided into four dynamic subpopulations (Fig. 1), which can be described with the following parameters of transmission rates:^1,10,17,21,24^

• $S\left(t\right)$ denotes the susceptible population.
• $E\left(t\right)$ is the exposed population who are infected but who have not been detected by testing.
• $I\left(t\right)$ denotes the people confirmed to have been infected and under treatment.
• $R\left(t\right)$ is the population living in a secure zone or not affected by COVID-19.
• *β* > 0 is the transmission rate from a susceptible population to an infected population, which has not been detected.
• Λ > 0 comprises new births and new residents per unit value of time.
• *μ* > 0 is the rate of natural death.
• *γ* > 0 is the transmission rate of confirmed infected people from the exposed population (1/*γ* is approximately the duration of the latent period).
• *δ* > 0 is the transmission rate of recovery from the exposed population (1/*δ* is approximately the duration for which infection is suspected).
• *α* > 0 is the transmission rate of recovery from the infected population (mean time spent in the “infectious” category of 1/*α*).

We can define the total population size by $N\left(t\right)$ as follows:^1,24^

$$N\left(t\right)=S\left(t\right)+E\left(t\right)+I\left(t\right)+R\left(t\right).$$ (1)

According to the inflows and outflows in Fig. 1, we can convert them into first-order, ordinary non-linear differential equations as follows:^24^

$$\frac{dS\left(t\right)}{dt}=\Lambda -\beta S\left(t\right)I\left(t\right)-\mu S\left(t\right),$$ (2)

$$\frac{dE\left(t\right)}{dt}=\beta S\left(t\right)I\left(t\right)-\varepsilon_{1}E\left(t\right),$$ (3)

$$\frac{dI\left(t\right)}{dt}=\gamma E\left(t\right)-\varepsilon_{2}I\left(t\right),$$ (4)

$$\frac{dR\left(t\right)}{dt}=\delta E\left(t\right)+\alpha I\left(t\right)-\mu R\left(t\right),$$ (5)

where $\beta S\left(t\right)I\left(t\right)$ is the number of individuals who become infected per unit of time and $\varepsilon_{1}=\left(\gamma +\mu +\delta \right)$, $\varepsilon_{2}=\left(\alpha +\mu \right)$, and *δ* = 0 lead to the usual SEIR model.^24^

Theorem 1 (***Solutions are never negative)***

All the solutions of the proposed SEIR model with its initial condition are a subset in the interval [0, *∞*) and $\left\{S\left(t\right),E\left(t\right),I\left(t\right),R\left(t\right)\right\}\geq 0\frac{x-\mu }{\sigma }$ for all values 0 ≤ *t* < *∞*.

Proof All the right-hand sides of the proposed SEIR model are completely continuous and locally Lipschitzian on $R_{+}^{4}$. The solutions $\left\{S\left(t\right),E\left(t\right),I\left(t\right),R\left(t\right)\right\}$ with the initial conditions exist and are unique in the interval $\left.\left[0,\infty \right.\right)$.^24^

From Eq. (2) where $\left[\Lambda -\beta S\left(t\right)I\left(t\right)\right]\geq 0$, we obtain the following valid inequality:

$$\frac{dS\left(t\right)}{dt}\geq -\mu S\left(t\right).$$ (6)

By solving the above differential inequality, we get

$$S\left(t\right)\geq S\left(0\right)e^{-\mu t}\geq 0.$$ (7)

Hence, $S\left(t\right)$ is a non-negative function for all values $t\in \left[0,\infty \right)$.

From Eq. (3), we have

$$\frac{dE\left(t\right)}{dt}\geq -\varepsilon_{1}E\left(t\right),$$ (8)

which gives

$$E\left(t\right)\geq E\left(0\right)e^{-\varepsilon_{1}t}\geq 0.$$ (9)

Hence, $E\left(t\right)$ is a non-negative function for all values $t\in \left[0,\infty \right)$.

In a similar manner for the remaining equations, we have

$$\frac{dI\left(t\right)}{dt}\geq -\varepsilon_{2}I\left(t\right)\to I\left(t\right)\geq I\left(0\right)e^{-\varepsilon_{2}t}\geq 0$$ (10)

and

$$\frac{dR\left(t\right)}{dt}\geq -\mu R\left(t\right)\to R\left(t\right)\geq R\left(0\right)e^{-\mu t}\geq 0.$$ (11)

Hence, $I\left(t\right)and\;R\left(t\right)$ are non-negative functions for all values of $t\in \left[0,\infty \right)$, which completes the proof.

Theorem 2 (Solutions domain)

All the solutions of the proposed SEIR model structure that initiate in $R_{+}^{4}$ are bound within the region *ψ* defined by $\psi =\left\{\left(S,E,I,R\right)\in R_{+}^{4}:\;0\leq N\left(t\right)\leq \frac{\Lambda }{\mu }\right\}$ as *t* → *∞*.

Proof. By differentiating both sides of Eq. (1), we get

$$N^{\prime }\left(t\right)=S^{\prime }\left(t\right)+E^{\prime }\left(t\right)+I^{\prime }\left(t\right)+R^{\prime }\left(t\right).$$ (12)

Substituting from the proposed SEIR model, we get

$$N^{\prime }=\Lambda -\mu N.$$ (13)

Then, we obtain

$$N\left(t\right)=\frac{\Lambda }{\mu }+\left(N\left(0\right)-\frac{\Lambda }{\mu }\right)e^{-\mu t}.$$ (14)

Thus, when *t* → *∞*, we get the solution $N\left(t\right)\subset \left[0,\frac{\Lambda }{\mu }\right]$, which completes the proof.^24^

### Epidemic equilibrium of the proposed SEIR model

To determine the epidemic equilibrium of this model, we set all the derivatives equal to zero and solved the system as follows:^10,22–24^

$$S^{\prime }\left(t\right)=E^{\prime }\left(t\right)=I^{\prime }\left(t\right)=R^{\prime }\left(t\right)=0\to \left\{S,E,I,R\right\}\equiv constants\neq 0.$$ (15)

Then, Eqs. (2)–(5) give

0=Λ−βSI−μS, (16)

$$0=\beta SI-\varepsilon_{1}E,$$ (17)

$$0=\gamma E-\varepsilon_{2}I,$$ (18)

0=δE+αI−μR. (19)

From Eq. (18), we have

$$E=\frac{\varepsilon_{2}}{\gamma }I.$$ (20)

From Eq. (17), we have

$$S=\frac{\varepsilon_{1}\varepsilon_{2}}{\beta \gamma }.$$ (21)

Substituting Eqs. (20) and (21) into Eq. (16), we get

$$I=\frac{\mu }{\beta }\left(\frac{\beta \gamma \Lambda }{\mu \varepsilon_{1}\varepsilon_{2}}-1\right)\neq 0,$$ (22)

where

$$R_{0}=\frac{\beta \gamma \Lambda }{\mu \varepsilon_{1}\varepsilon_{2}}=\frac{\beta \gamma \Lambda }{\mu \left(\gamma +\mu +\delta \right)\left(\alpha +\mu \right)}.$$ (23)

The number $R_{0}$ is the RN.^10,22–24^

This formula is in agreement with the standard formula of the RN of SEIR when *δ* = 0. The RN is positive, and it is zero if there is no transmission, where *β* = 0.0, and it can be interpreted as the number of secondary cases or new infection rate.

### Equilibrium by application of the Jacobian matrix

To obtain $R_{0}$ by using the Jacobian matrix, we consider that the disease-free equilibrium (DFE) of the proposed SEIR model is acquired by setting *E* = *I* = *R* = 0 in Eqs. (16)–(19). Hence, we obtain the DFE in the form $E_{0}=\left(\frac{\Lambda }{\mu },0,0,0\right)$.^24^

The Jacobian matrix of the proposed SEIR model takes the following form:

$$J_{E_{0}}=\left[\begin{array}{cccc} -\beta I-\mu & 0 & -\beta S & 0\\ \beta I & \varepsilon_{1} & \beta S & 0\\ 0 & \gamma & -\varepsilon_{2} & 0\\ 0 & \delta & \alpha & -\mu \end{array}\right].$$ (24)

Using the Jacobian linearization method, we linearize the first two equations considering the system $I=0\;,\;E=0,\;\;and\;\;S=\frac{\Lambda }{\mu }$.

Hence, we consider the following functions:

$$F\left(S,I\right)=\Lambda -\beta S\left(t\right)I\left(t\right)-\mu S\left(t\right),$$ (25)

$$G\left(S,I\right)=\beta S\left(t\right)I\left(t\right).$$ (26)

Then, we have

$$\left[\begin{array}{cc} F_{S} & F_{I}\\ G_{S} & G_{I} \end{array}\right]\left[\begin{array}{c} S\left(t\right)-S\left(0\right)\\ I\left(t\right)-I\left(0\right) \end{array}\right]=\left[\begin{array}{cc} -\beta I\left(0\right)-\mu & -\beta S\left(0\right)\\ \beta I\left(0\right) & \beta S\left(0\right) \end{array}\right]\left[\begin{array}{c} S\left(t\right)-S\left(0\right)\\ I\left(t\right)-I\left(0\right) \end{array}\right].$$ (27)

By substituting from the equilibrium position, we obtain

$$\left[\begin{array}{c} S^{\prime }\left(t\right)\\ E^{\prime }\left(t\right) \end{array}\right]=\left[\begin{array}{cc} -\mu & -\frac{\Lambda \beta }{\mu }\\ 0 & \frac{\Lambda \beta }{\mu } \end{array}\right]\left[\begin{array}{c} S\left(t\right)-\frac{\Lambda }{\mu }\\ I\left(t\right) \end{array}\right]+\left[\begin{array}{c} 0\\ -\varepsilon_{1}E\left(t\right) \end{array}\right].$$ (28)

Hence, the coupled non-linear equations (2) and (3) have been linearized to be in the following forms:

$$\frac{dS\left(t\right)}{dt}=\Lambda -\mu S\left(t\right)-\frac{\beta \Lambda }{\mu }I\left(t\right)$$ (29)

and

$$\frac{dE\left(t\right)}{dt}=-\varepsilon_{1}E\left(t\right)+\frac{\Lambda \beta }{\mu }I\left(t\right).$$ (30)

Hence, the Jacobian matrix of the proposed SEIR model after linearization at equilibrium is given by^24^

$$J_{E_{0}}=\left[\begin{array}{cccc} -\mu & 0 & -\frac{\beta \Lambda }{\mu } & 0\\ 0 & -\varepsilon_{1} & \frac{\beta \Lambda }{\mu } & 0\\ 0 & \gamma & -\varepsilon_{2} & 0\\ 0 & \delta & \alpha & -\mu \end{array}\right].$$ (31)

### Uniqueness of the equilibrium condition

If the matrix $J_{E_{0}}$ is obtained from linearization and is the Jacobian evaluated at equilibrium $DFE\left(E_{0}\right)=\left(\frac{\Lambda }{\mu },0,0,0\right)$, the condition $\left|J_{E_{0}}\right|\neq 0$ means that the equilibrium is isolated, so there is a disk around it that does not contain other equilibria.

Hence, from (31), we have

$$\begin{array}{ll} \det\left(J_{E_{0}}\right) & =\left|\begin{array}{cccc} -\mu & 0 & -\frac{\beta \Lambda }{\mu } & 0\\ 0 & -\varepsilon_{1} & \frac{\beta \Lambda }{\mu } & 0\\ 0 & \gamma & -\varepsilon_{2} & 0\\ 0 & \delta & \alpha & -\mu \end{array}\right|=\mu^{2}\;\varepsilon_{1}\;\varepsilon_{2}\left(\frac{\beta \;\gamma \;\Lambda }{\mu \;\varepsilon_{1}\;\varepsilon_{2}}-1\right)\\ & =\mu^{2}\;\varepsilon_{1}\;\varepsilon_{2}\left(R_{0}-1\right)\neq 0. \end{array}$$ (32)

Thus, condition (22) is the only condition of the equilibrium of the proposed SEIR model.

Therefore, the unique equilibrium condition of the proposed SEIR model is

$$\frac{\beta \;\gamma \;\Lambda }{\mu \;\varepsilon_{1}\;\varepsilon_{2}}-1\neq 0.$$ (33)

Hence, the RN $R_{0}=\frac{\beta \;\gamma \;\Lambda }{\mu \;\varepsilon_{1}\;\varepsilon_{2}}$ is also unique.^24^

Theorem 3 (Stability analyses of DFE)

The proposed SEIR model $DFE\left(E_{0}\right)=\left(\frac{\Lambda }{\mu },0,0,0\right)$ is locally asymptotically stable under the condition $R_{0}<1$ and unstable if $R_{0}>1$.

Proof. From the Jacobian matrix of the system (31), which is defined at $DFE\left(E_{0}\right)=\left(\frac{\Lambda }{\mu },0,0,0\right)$, and by calculating the characteristic equation, which is given by $\left|J_{E_{0}}-\lambda I_{4}\right|=0$, where *λ* is the eigenvalues parameter and *I*_4_ is the identity matrix of order 4, we have two roots *λ*_1_ = *λ*_2_ = −*μ*, and the remaining roots are the solution to the following equation:

$$\left|\begin{array}{cc} -\varepsilon_{1}-\lambda & \frac{\beta \Lambda }{\mu }\\ \gamma & -\varepsilon_{2}-\lambda \end{array}\right|=0,$$ (34)

which gives

$$\left(\varepsilon_{1}+\lambda \right)\left(\varepsilon_{2}+\lambda \right)-\frac{\gamma \beta \Lambda }{\mu }=0.$$ (35)

The roots of the above equation after inserting $R_{0}$ take the following forms:

$$\begin{array}{ll} \lambda_{3} & =-\frac{1}{2}\left(\varepsilon_{1}+\varepsilon_{2}-\sqrt{{\left(\varepsilon_{1}-\varepsilon_{2}\right)}^{2}+4\varepsilon_{1}\varepsilon_{2}R_{0}}\right),\\ \lambda_{4} & =-\frac{1}{2}\left(\varepsilon_{1}+\varepsilon_{2}+\sqrt{{\left(\varepsilon_{1}-\varepsilon_{2}\right)}^{2}+4\varepsilon_{1}\varepsilon_{2}R_{0}}\right). \end{array}$$ (36)

Now, we have the following situations:

$$\left\{\begin{array}{cc} R_{0}>1\to & \lambda_{3}>0,\lambda_{4}<0\\ R_{0}=1\to & \lambda_{3}=0,\lambda_{4}<0\\ R_{0}<1\to & \lambda_{3}<0,\lambda_{4}<0 \end{array}\right\}.$$ (37)

Thus, if $R_{0}<1$, then the DFE *E*_0_ is locally asymptotically stable. If $R_{0}\geq 1$, then the DFE *E*_0_ is locally asymptotically unstable.^24^

### Local sensitivity analysis of RN $\left(R_{0}\right)$

Local sensitivity analysis examines the change in the output values that result from a change in one input value (one parameter).

The sensitivity or elasticity of quantity *H* concerning the parameter p is given by^24^

$${℘}_{H}^{p}=\frac{\partial H}{\partial p}/\frac{H}{p}=\pm \frac{\%\Delta H}{\%\Delta p}.$$ (38)

The sensitivity of *H* with respect to *p* is positive if *H* is increasing with respect to p and negative if *H* is decreasing with respect to p.

Applying formula (38) into $R_{0}$ takes the following form:

$$R_{0}=\frac{\beta \gamma \Lambda }{\mu \left(\gamma +\mu +\delta \right)\left(\alpha +\mu \right)}.$$ (39)

Then,

$${℘}_{R_{0}}^{\beta }=\frac{\partial R_{0}}{\partial \beta }/\left(\frac{R_{0}}{\beta }\right)=1>0,$$ (40)

$${℘}_{R_{0}}^{\gamma }=\frac{\partial R_{0}}{\partial \gamma }/\left(\frac{R_{0}}{\gamma }\right)=\frac{\mu +\delta }{\varepsilon_{1}}>0,$$ (41)

$${℘}_{R_{0}}^{\mu }=\frac{\partial R_{0}}{\partial \mu }/\left(\frac{R_{0}}{\mu }\right)=-\left(1+\frac{\left(\varepsilon_{1}+\varepsilon_{2}\right)\mu }{\varepsilon_{1}\varepsilon_{2}}\right)<0,$$ (42)

$${℘}_{R_{0}}^{\delta }=\frac{\partial R_{0}}{\partial \delta }/\left(\frac{R_{0}}{\delta }\right)=-\frac{\delta }{\varepsilon_{1}}<0,$$ (43)

$${℘}_{R_{0}}^{\alpha }=\frac{\partial R_{0}}{\partial \alpha }/\left(\frac{R_{0}}{\alpha }\right)=-\frac{\alpha }{\varepsilon_{2}}<0.$$ (44)

Hence, a 1% increase in each one $\left(\mu ,\delta ,\alpha \right)$ will produce a $\left(1+\frac{\left(\varepsilon_{1}+\varepsilon_{2}\right)\mu }{\varepsilon_{1}\varepsilon_{2}},\frac{\delta }{\varepsilon_{1}},\frac{\alpha }{\varepsilon_{2}}\right)\%$ decrease in $R_{0}$, whereas a 1% increase in *γ* will produce an $\left(\frac{\mu +\delta }{\varepsilon_{1}}\right)\%$ increase in $R_{0}$. From Eq. (40), ${℘}_{R_{0}}^{\alpha }=1$ means that a 1% increase *α* will produce a rise of 1% in $R_{0}$.^24^

### Global stability of equilibria of the SEIR model (Lyapunov stability theorem)

Lyapunov functions are scalar functions that can be used to prove the global stability of equilibrium. Lyapunov stated that if a function *V*(*x*) is globally positively definite and radially unbounded, and its time derivative is globally negative, *V*(*x*) < 0 *for all x* ≠ x^*^, then the equilibrium x^*^ is globally stable for the autonomous system $x^{\prime }=f\left(x\right)$, and $V\left(x\right)$ is called the Lyapunov function.^24^

Theorem 4 (Global stability)

The SEIR model $DFE\left(E_{0}\right)=\left(\frac{\Lambda }{\mu },0,0,0\right)$ is globally stable of the DFE under the condition $R_{0}<1$.

Proof. We will consider the proposed SEIR model on the space of the first three variables only $\left(S,E,I\right)$. It is clear that if the DFE for the first three equations is globally stable, then *R* → 0, and the DFE for the full SEIR model is globally stable.

We construct the Lyapunov function on $R_{+}^{3}$ in the following form:^24^

$$V=\kappa \left(S-S^{*}-S^{*}\ln\left(\frac{S}{S^{*}}\right)\right)+\frac{E}{\varepsilon_{2}}+\frac{I}{\gamma },$$ (45)

where *κ* is a parameter that will be determined later and $S^{*}=\frac{\Lambda }{\mu }$.

Equation (45) shows that, at the DFE $\left(S^{*}=\frac{\Lambda }{\mu },0,0\right)$, *V* = 0.

Now, we have to show that *V* > 0 for all $\left(S,E,I\right)\geq \left(\frac{\Lambda }{\mu },0,0\right)$.

Equation (45) can be re-written as

$$V=\kappa S^{*}\left(\frac{S}{S^{*}}-1-\ln\left(\frac{S}{S^{*}}\right)\right)+\frac{E}{\varepsilon_{1}}+\frac{I}{\gamma }.$$ (46)

The first term is positive for any value of *S*/S^*^, and the remaining two terms are also non-negative, so *V* > 0.

Now, taking the derivative of Eq. (45), we obtain

$$V^{\prime }=\kappa \left(1-\frac{S^{*}}{S}\right)S^{\prime }+\frac{E^{\prime }}{\varepsilon_{1}}+\frac{I^{\prime }}{\gamma }.$$ (47)

Substituting the first three equations of the SEIR model and using Eq. (21), we obtain

$$V^{\prime }=2\Lambda \kappa -\beta \kappa SI-\mu \kappa S-\frac{\kappa \Lambda^{2}}{\mu S}+\frac{\kappa \Lambda }{\mu }\beta I+\frac{\beta }{\varepsilon_{1}}SI-\frac{\varepsilon_{2}}{\gamma }I.$$ (48)

We choose $\kappa =\frac{1}{\varepsilon_{1}}$, then we have

$$V^{\prime }=-\frac{\Lambda }{\varepsilon_{1}}\left(\frac{\mu S}{\Lambda }+\frac{\Lambda }{\mu S}-2\right)+\frac{\varepsilon_{2}}{\gamma }I\left(R_{0}-1\right),$$ (49)

$R_{0}<1$, so the last term is non-positive.

For the first term, consider $\frac{\mu S}{\Lambda }=z$, then the term within the brackets takes the form $\left(z+\frac{1}{z}-2\right)=\frac{{\left(z-1\right)}^{2}}{z}>0$, which gives two possibilities. The first one is at the equilibrium point $S=S^{*}=\frac{\Lambda }{\mu }$, which leads to *z* = 1. Then, the first term vanishes completely. Hence, we have the final term only, and it is non-negative. Thus, *V*′ < 0.

The second possibility is *x* ≠ 1, then the two terms are non-positive. Thus, *V*′ < 0.

Therefore, *V*′ < 0 for every $\left(S\left(t\right),E\left(t\right),I\left(t\right)\right)\;\;\geq \left(\frac{\Lambda }{\mu },0,0\right)$.

According to the Lyapunov theorem, the DFE is globally asymptotically stable for the system of the proposed SEIR model.^24^

### Solutions for the system of the proposed SEIR model

We assume that the initial conditions of the system (2)–(5) take the following form:

$${\left.\left\{S\left(t\right),E\left(t\right),I\left(t\right),R\left(t\right)\right\}\right|}_{t=0}=\left\{S\left(0\right),E\left(0\right),I\left(0\right),R\left(0\right)\right\}.$$ (50)

By using MAPLE software, we solved the system, then we have

$$I\left(t\right)=\left[I\left(0\right)\cosh\left(\frac{\omega }{2\mu }\;t\right)+\frac{2\varpi \mu }{\omega }\sinh\left(\frac{\omega }{2\mu }\;t\right)\right]e^{-\;\;\frac{\left(\varepsilon_{1}+\varepsilon_{2}\right)}{2}\;t},$$ (51)

where $\omega =\sqrt{\mu {\left(\varepsilon_{1}^{2}-\varepsilon_{2}^{2}\right)}^{2}+4\Lambda \gamma \beta }$, $\varpi =\gamma E\left(0\right)+\frac{\left(\varepsilon_{1}-\varepsilon_{2}\right)}{2}I\left(0\right)$.

We make our scope only on the infection function $I\left(t\right)$, and the remaining function can be obtained by substituting Eq. (51) into the system.

## RESULTS

### Real situation

The specific evidence for the COVID-19 epidemic in Saudi Arabia was tested. By 3 March 2020, COVID-19 had spread to Saudi Arabia. A low number of COVID-19 cases were identified until 1 April 2020 after which the number of cases was reported to increase. Therefore, we considered 1 April 2020 as the real start of the COVID-19 epidemic in Saudi Arabia.^25^

We used tables of statistics issued from the Saudi Ministry of Health to obtain more information about the population, mortality rate, and population growth rate in Saudi Arabia.^26^ We also assessed the daily official statement issued by the Saudi Ministry of Health, as well as Wikipedia^TM^^27^ (which also uses data from the Saudi Ministry of Health).

**FIG. 2.**
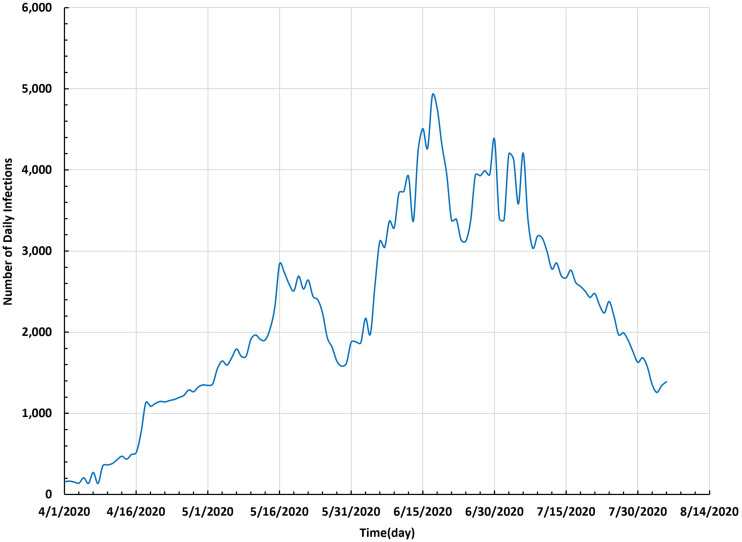
Number of daily infections in Saudi Arabia between 1 April and 5 August 2020.

**FIG. 3.**
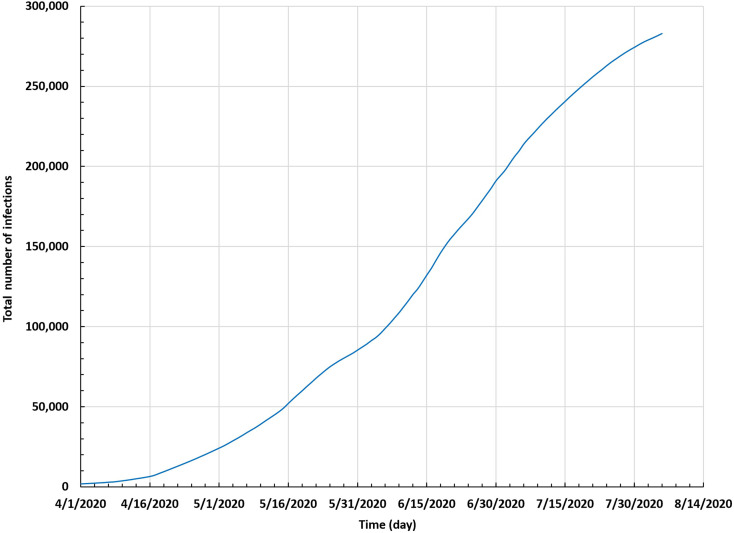
Total number of infections in Saudi Arabia between 1 April and 5 August 2020.

To study the spread of COVID-19 in Saudi Arabia up to 5 August 2020, we represented the curve of the number of daily infections and the time-series curve of the total number of infections, as shown in Figs. 2 and 3, respectively.

Figure 2 shows that the number of infections on 1 April 2020 was 157. The number of infections reached a peak (4919) on 17 June 2020. After this date, the number of daily infections decreased up to 5 August 2020.

Figure 3 shows that the total number of infections during the same interval started with 157 infections and reached an accumulated number of 282 824 infections on 5 August 2020.^26^ We used these data through the SEIR model to discern whether there was a convergence between the modeling results and real data.

### Application of the modified SEIR model using Saudi Arabia data

We divided verification of the modified SEIR model into two stages. The first stage consisted of applying the real data of COVID-19 spread in the interval between 1 April 2020 and the peak position on 17 June 2020. The second stage consisted of applying the real data of COVID-19 spread in the interval between 18 June 2020 and 5 August 2020.

For the first stage and according to the official data for Saudi Arabia,^26^ we obtained the total population in Saudi Arabia on 17 June 2020 taking the value $S\left(0\right)=34,218,200$. The total number of the exposed population who had become infected was assumed to be $E\left(0\right)=1.0\times 10^{3}$, whereas the number of infections was $I\left(0\right)=157$. The number of people who recovered in this population at the same time was $R\left(0\right)=100$. The total number of new births in Saudi Arabia and new residents was Λ ≈ 2300 persons/day. The rate of natural deaths was ∼1030 persons/day, which gave *μ* ≈ 3 × 10^−5^. The other parameters were assumed according to the real situation (Table I).

**TABLE I.** Values of parameters.^24,26^

Parameter	Value
*γ*	0.2
*δ*	0.1
*α*	0.03

After using the parameter values shown in Table I and MAPLE software, we obtained results indicating the number of daily infections as outcomes of the modified SEIR model. The value of the parameter *β* (rate of transmission from the susceptible population to an infected population in Saudi Arabia) within the interval mentioned above was *β* = 1.18 × 10^−9^. Moreover, $R_{0}=2.008>1$, that is, the transmission rate at which the susceptible individual became an exposed individual was >1, which meant that the spread of COVID-19 was not stable in this period.

In the second stage and according to the official data of Saudi Arabia, we considered a new initial state of the system based on the modified SEIR model. The number of infections $I\left(0\right)=4757$, the value of the parameter *β* within the interval mentioned above was *β* = 1.12 × 10^−9^, and the other parameters are shown in Table II. Moreover, $R_{0}=0.596<1$. Hence, the transmission rate at which the susceptible individual became an exposed individual was <1, which meant that the spread of COVID-19 in this period was stable.

**TABLE II.** Values of parameters.

Parameter	Value
*γ*	0.1
*δ*	0.14
*α*	0.06

**FIG. 4.**
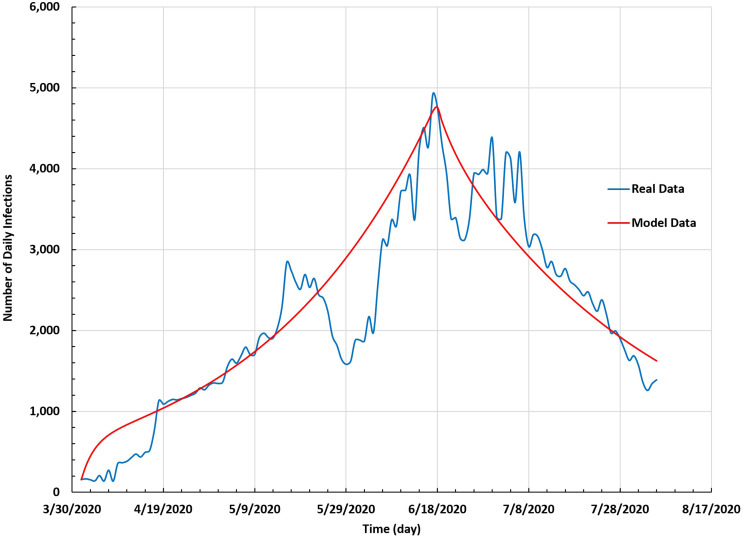
Number of daily infections based on the SEIR model against the real data in Saudi Arabia between 1 April and 5 August 2020.

The convergence between the results from the modified SEIR model and real data is displayed in Fig. 4. The latter shows the number of daily infections based on the modified SEIR model against the actual data in Saudi Arabia between 1 April 2020 and 5 August 2020. The curve that represents the results of the modified SEIR model works as a trend to the real-data curve. Thus, the results obtained from the modified SEIR model converged with the actual data.

**FIG. 5.**
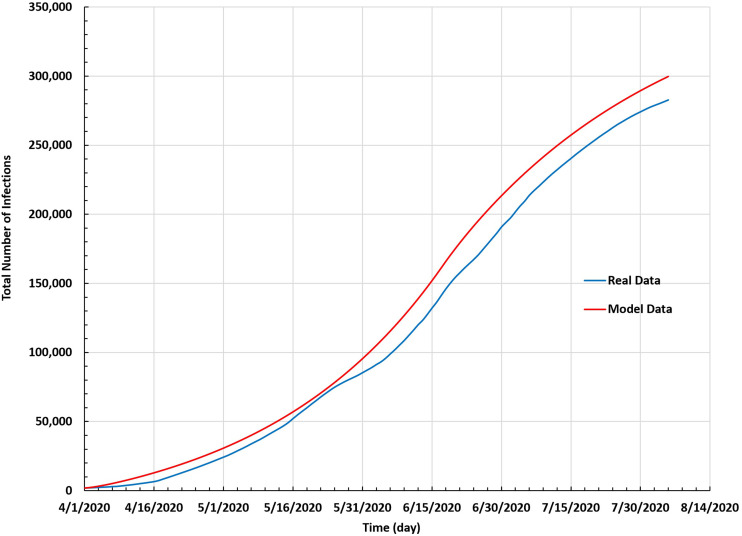
Total number of infections based on the SEIR model against the real data in Saudi Arabia between 1 April and 5 August 2020.

The convergence between the results of the proposed SEIR model and real data is displayed in Fig. 5. The latter shows the total number of infections based on the modified SEIR model against the actual data in Saudi Arabia between 1 April and 5 August 2020. The two curves are aligned closely and display similar behavior.

Figures 3 and 4 show that the results from the modified SEIR model were close to the real data: this model was successful.

### Predicting how COVID-19 will spread in Saudi Arabia in the next 60 days

Next, we predicted the spread of COVID-19 in Saudi Arabia based on current data and parameters with the same rates without any change in procedures. We illustrated the results of the number of daily infections by applying the modified SEIR model for the next 60 days from 18 June to 4 October 2020.

**FIG. 6.**
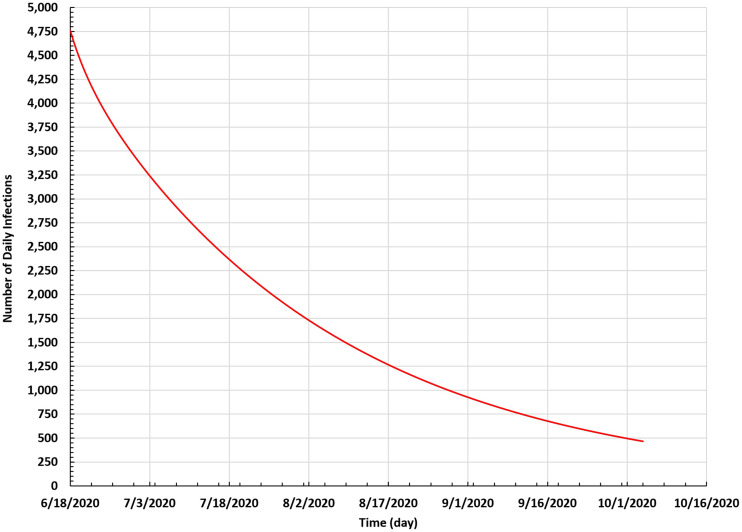
Number of daily infections based on the SEIR model in Saudi Arabia between 18 June and 4 October 2020.

Figure 6 shows that the number of infections will decrease, and the spread of COVID-19 will continue in a stable manner. Moreover, the number of daily infections will be <500 cases on 4 October 2020.

## DISCUSSION

### The “ideal” protocol to reduce the spread of COVID-19 in Saudi Arabia

To realize the ideal situation, which can help reduce the spread of COVID-19 in Saudi Arabia, we must start implementing the following four main protocols and procedures:

1. Decrease the value of the transmission rate from the susceptible population to the population that is infected but which has not been detected by testing the population in the interval *β* ≤ 1.12 × 10^−9^ (prevention is better than treatment).
2. Decrease the transmission rate of people confirmed to be infected from the exposed population *γ* < 0.2 to increase the duration of the latent period. This can be achieved by ensuring that the infected population stays away for an extended time from other populations and stays in secure zones.
3. Increase the transmission rate of recovery from the exposed population *δ* > 0.1. This strategy involves reducing the duration of suspicion of infection using practical tools and methods to discover the cases of confirmed infection faster.
4. Increase the transmission rate of recovery from the infected population *α* > 0.03. This can be achieved by reducing the time spent in the “infectious” category using efficacious treatment and supplying the population with vitamins, tonics, and supplements.

## CONCLUSIONS

We constructed a modified SEIR model for the outbreak of COVID-19. This model is a modified approach for evaluation and management of the COVID-19 pandemic. The real data of COVID-19 spread in Saudi Arabia were used to verify the results of our modified SEIR model. We demonstrated that the modified SEIR model could be employed to assess the spread of epidemics such as COVID-19 in Saudi Arabia and other countries.

The ideal protocol consists of four steps. Advice has been introduced (in detail) to help the Saudi Arabia population slow the spread of COVID-19. One of the main concepts is that prevention is better than treatment.

The other essential issues that help to slow the spread of COVID-19 are to stay at home as much as possible and for infected people to remain in an isolated zone or a secure area. Finally, we must offer suitable treatment for those infected with SARS-CoV-2 and supply non-infected people with vitamins, tonics, and supplements to protect them.

## AUTHORS’ CONTRIBUTIONS

H.M.Y., M.A.E., and N.A.A. conceived the original idea and led the overall study. A.A.E. and A.M.S. wrote this manuscript and revised it carefully. H.M.Y, M.A.E, N.A.A., A.A.E., and A.M.S. collected and analyzed all data. All authors approved the final version of this manuscript.

## Data Availability

The datasets analyzed during the current study are available in the following repositories:1. Saudi Ministry of Health (www.moh.gov.sa/en/Pages/default.aspx], 2. COVID-19 in Saudi_Arabia (https://en.wikipedia.org/wiki/COVID-19_pandemic_in_Saudi_Arabia), and 3. Saudi Center for Diseases Prevention and Control (https://covid19.cdc.gov.sa/ar/).
